# First isolation, identification, and pathogenicity evaluation of an EV-G6 strain in China

**DOI:** 10.3389/fvets.2024.1431180

**Published:** 2024-07-24

**Authors:** Pei Zhu, Zhan-Hong Li, Zhuo-Ran Li, Zhen-Xing Zhang, Jian-Ling Song

**Affiliations:** ^1^Yunnan Tropical and Subtropical Animal Virus Diseases Laboratory, Yunnan Animal Science and Veterinary Institute, Kunming, China; ^2^Key Laboratory of Transboundary Animal Diseases Prevention and Control (Co-Construction by Ministry and Province), Yunnan Animal Science and Veterinary Institute, Kunming, China

**Keywords:** enterovirus G, genotype 6, virus isolation, phylogenetic analysis, pathogenicity

## Abstract

Enterovirus G (EV-G) belongs to the Picornaviridae family and infects porcine populations worldwide. A total of 20 EV-G genotypes (EV-G1 to EV-G20) have been identified. In this study, we isolated and characterized an EV-G strain, named EV-G/YN29/2022, from the feces of diarrheic pigs. This was the first EV-G6 strain isolated in China. Comparison of the whole genome nucleotide and corresponding amino acid sequences showed that the isolate was more closely related to those of the EV-G6 genotype than other genotypes, with the complete genome sequence similarity ranging from 83.7% (Iba46442) to 84.4% (PEV-B-KOR), and corresponding amino acid homology ranged from 96% (Iba46442) to 96.8% (PEV-B-KOR). Similarly, the VP1 gene and corresponding amino acid sequences of EV-G/YN29/2022 were highly similar to those of the EV-G6 genotype (>82.9% and >94.3%, respectively). Thus, the isolated strain was classified as EV-G6 genotype. This was the first EV-G6 strain isolated in China. Pathogenicity analyses revealed that EV-G/YN29/2022 infection caused mild diarrhea, typical skin lesions, and weight reduction. The strain was mainly distributed to the intestinal tissue but was also found in the brain, mesenteric lymph nodes, spleen, and liver. Our results can be used as a reference to further elucidate the epidemiology, evolution, and pathogenicity of EV-G.

## Introduction

Porcine enteroviruses (PEVs), members of the Picornaviridae family, were initially divided into 13 serotypes (PEV-1 to -13) based on virus neutralization assays. These serotypes were subdivided into three groups (CPE-I, -II, and -III) based on the differences in their cytopathic effect (CPE). CPE-I comprised PEV-1 to -7 and PEV-11 to -13, CPE-II comprised PEV-8, and CPE-III comprised PEV-9 and -10. Eventually, with the availability of the full genome sequences of these serotypes, the International Committee on Taxonomy of Viruses (ICTV) reclassified these serotypes based on their phylogeny and CPE differences. Thus, PEV-1 to -7 and PEV-11 to -13 were classified as porcine teschovirus (PTV), PEV-8 was classified as porcine sapelovirus (PSV), and PEV-9 and -10 were classified as enterovirus G (EV-G) (1–4).

The EV-G genome comprises a 7,400–7,500 nucleotides long, positive-sense, single-stranded RNA. It includes only one open reading frame (ORF) flanked by a 5′-untranslated region (UTR) and 3′-UTR with a poly(A) tail. The ORF encodes a polypeptide that is cleaved to produce four structural capsid proteins (VP1, VP2, VP3, and VP4) and seven nonstructural proteins (2A^pro^, 2B, 2C, 3A, 3B, 3C pro, and 3D^pol^) (1–3). VP1 gene sequence exhibits the highest variability to the other parts of the genome and is used to identify the various EV-G genotypes. A total of 20 genotypes (EV-G1 to EV-G20) have been identified worldwide (5, 6).

EV-G was first isolated from pigs presenting with atypical skin lesions (7, 8). Domestic pigs and wild boars of all ages are susceptible to EV-G (3). Generally, pigs with EV-G infection remain asymptomatic. However, the infection sometimes manifests as mild disease-like skin lesions. Previous studies on EV-G-infected piglets have reported neurological symptoms, such as flaccid paralysis of the hind limbs, circling, and abnormal excitement; pathological damage to the brain; and a significant decrease in the daily weight gain of infected piglets, suggesting high EV-G pathogenicity in piglets (8, 9). In recent years, a recombinant virus comprising EV-G and porcine torovirus (ToV), characterized by the insertion of papain-like cysteine protease gene of ToV (ToV-PLCP) into the EV-G genome, has emerged (8–11). The ToV-PLCP gene reportedly acts as an “innate immune antagonist,” suppressing the host cellular innate immune responses, allowing EV-G to acquire new infectious properties, and ultimately altering the EV-G pathogenicity (9).

Currently, EV-Gs have been found in the United States, Brazil, the United Kingdom, Germany, Hungary, Spain, the Czech Republic, China, Japan, Korea, and Vietnam (12–14). In China, EV-G was first isolated in Shanghai in 2012, followed by successive isolations from pigs in the Anhui, Jiangsu, and Guangxi provinces. To the best of our knowledge, EV-G has not yet been isolated from the Yunnan pigs. The present study reported the first isolation of an EV-G strain from pigs in the Yunnan province. Full genome sequencing showed that the isolate carried the EV-G6 genotype, providing a scientific reference for further epidemiological investigations of EV-G in the Yunnan pigs.

## Materials and methods

### Sample detection and cell culture

Fecal and intestinal tissue samples were collected from 32 pigs exhibiting diarrhea symptoms in the Yunnan province. The samples were stored at 4°C and sent to the Yunnan Tropical and Subtropical Animal Virus Diseases Laboratory for common diarrhea pathogen test and EV-G isolation. The samples were diluted five-fold in MEM. The mixtures were vortexed and centrifuged at 12,000× g at 4°C for 10 min. The supernatants were collected. The total viral RNA was extracted from the supernatants using the MiniBEST Viral RNA/DNA Extraction Kit Ver.5.0 (Takara Bio, Dalian, China). RT-PCR was used to detect porcine epidemic diarrhea virus (PEDV), transmissible gastroenteritis virus (TGEV), porcine rotavirus (PoRV), porcine delta coronavirus (PDCoV), and EV-G using the PrimeScript™ One Step RT-PCR Kit (Takara Bio). The supernatants containing the EV-G-positive samples were filtered through a 0.22-μm filter (Millipore, Billerica, MA, United States) and stored at −80°C until EV-G isolation.

Baby hamster kidney (BHK-21) cells (The China Center for Type Culture Collection, Wuhan, China) were cultured at 37°C in Modified Eagle’s Medium (MEM; Gibco, Invitrogen, CA, United States) supplemented with 8% heat-inactivated fetal bovine serum (FBS; Gibco), 0.1 mg/mL streptomycin, and 100 U/mL penicillin.

### Virus isolation

EV-G-positive samples were passed through 0.22-μm filters (Millipore, Billerica, MA, United States). Then, the 25 cm^2^ monolayer of BHK-21 cells was incubated in 1 mL of the filtrate till 90% confluency. Then, the cells were washed thrice with phosphate-buffered saline (PBS) and cultured in 10 mL of MEM supplemented with antibiotics (100 units/mL of penicillin and100 μg/mL of streptomycin). The culture was incubated at 37°C, and the CPE on the cells was assessed daily. The culture supernatants were collected after at least five blind passages until an obvious CPE was observed. To obtain the cell pellets, the culture was subjected to three freeze–thaw cycles and centrifuged at 8,000× g for 10 min at 4°C. The supernatants were stored at −80°C for EV-G detection. The viral nucleic acids were extracted from 200 μL supernatant and subjected to EV-G-specific RT-PCR to identify the EV-G RNA. The PCR products were sequenced for further confirmation.

### Virus purification and replication kinetics analysis

The isolated virus was purified via plaque purification. Briefly, BHK-21 cells were cultured in 12-well plates. The growth medium was then discarded at 90% confluency. Next, the cells were incubated with 10-fold serially diluted (10^−2^ to 10^−6^) stock virus solution (0.4 mL/well) for 1 h at 37°C. Then, the virus inoculum was discarded, and the plates were washed twice with PBS. Then, the cells were inoculated with 2 mL MEM supplemented with 1% (w/v) agarose (Sigma-Aldrich, St. Louis, MO, United States) and 1% fetal bovine serum (FBS; Gibco) and incubated at 37°C for 96 h. Next, 1.5 mL of 0.01% neutral red staining solution (Solarbio, Beijing, China) was added per well. Then, a clear and uniform plaque was chosen to inoculate a fresh BHK-21 cell culture. When CPE was observed, the cells were freeze-thawed twice and centrifugated at 8,000× g for 10 min. The supernatant was subjected to at least three additional rounds of plaque purification until the plaques were of similar size and clear morphology. Finally, purified cultured virus samples were obtained.

The titer of the purified virus samples was determined using the etch-a-sketch method as previously described. Then, the BHK-21 cells were infected with the isolated strain at 0.01 multiplicity of infection (MOI). After adsorption at 37°C for 60 min, the cells were inoculated with MEM supplemented with antibiotics (100 units/mL of penicillin and 100 μg/mL of streptomycin). The plates were incubated at 37°C under 5% CO_2_. Culture supernatants were harvested in triplicates at 6,12, 24, 36, 48, 60, 72, 84, and 96 h post-infection (hpi) via centrifugation at 12,000× g for 10 min at 4°C and used for virus titration using the plaque assay. The virus samples were titrated thrice at each time point.

### Transmission electron microscopy

BHK-21 cells were infected with the isolated strain and cultured. The culture supernatants were collected when the CPE was >80% and centrifuged at 40,000 rpm at 4°C for 4 h. The cell pellet was re-suspended in 1 mL of TNE Buffer (Solarbio, Beijing, China) and stored at 4°C overnight. The samples were then negatively stained with 2% phosphotungstic acid and examined using a transmission electron microscope (Hitachi TEM system, Japan, HT7800).

### Virus genome amplification and sequencing

Virus RNA was extracted from the culture supernatants using Viral RNA Mini Kit (Takara Inc.) and reverse transcribed using TaKaRa One Step RT-PCR Kit with random primers. Five primer pairs were used to obtain the full genome sequence of the isolated EV-G strain (Table 1). The primers were designed using Oligo 7.0, with EV-G whole gene sequences (GenBank No. JQ818253, LC316820, LC316819) as reference sequences. In addition, 100–300 bp amplification overlap zones were set for neighboring primers in order to splice the whole genome sequence. Primers were synthesized by Shanghai Jierui Bioengineering Co.

**Table 1:** Primers used for amplifying the complete genome sequence of EV-G.

Primer	Sequence (5′~3′)	Position^a^	PCR product length (bp)
EV-G-F1	TTAAAACAGCCTGTGGGTTGTTCC	1–24	1,767
EV-G-R1	GTGGGGATTCCCTGAACAATGGCTTT	1,742–1,767	
EV-G-F2	CCTACATAAATTCAATTCCTATGG	1,581–1,604	1,628
EV-G-R2	CTCTAACGTGYTTTGGCTTGGCATA	3,185–3,209	
EV-G-F3	CCCTCAGTCTTCTTYCAAGCAAATGG	2,965–2,991	1,558
EV-G-R3	CAGAGTGTTCRTATTCTGCAAG	4,502–4,523	
EV-G-F4	AGCAACGTATTGAACCTGTCTG	4,416–4,437	1,397
EV-G-R4	GTTGGGAAGTTGTACATCATGGT	5,795–5,813	
EV-G-F5	AACCTCACCGATGAAGAGGGTGT	5,566–5,589	1,827
EV-G-R5	ACACCCCATCCGGTGGGTGTATTGA	7,369–7,393	

^a^According to the sequence of PEV-B-KOR (Acc. No. JQ818253).

The PCR protocol was as follows: Pre-denaturation at 94°C for 3 min, followed by 35 cycles of denaturation at 94°C for 30 s, annealing at 60°C for 30 s, and extension at 72°C for 90 s, and final extension at 72°C for 10 min. PCR products were resolved using agarose gel electrophoresis.

The target PCR products were purified using a MiniBEST Agarose Gel DNA Extraction Kit (TaKaRa). The purified fragments were then ligated into T-vector pMD18 (TaKaRa) and used to transform competent *Escherichia coli* DH5a. Then, the colonies were screened using respective fragment amplification primers, and two positive clones were selected for each fragment and sequenced by Shanghai Jierui Bioengineering Co.

### Sequence characterization and phylogenetic analysis

The full genome sequence of EV-G was assembled using the DNAMAN software. Next, the amino acid sequences corresponding to the genome were predicted using NCBI’s ORF analysis software. Genome characteristics were analyzed based on the size of the complete genome, UTRs and ORFs, length of each gene and its coding amino acid sequence, and percentage of different bases in the genome.

To analyze the phylogenetic relationship between the EV-G strain isolated in the present study and other EV-G strains, full genome sequences and corresponding amino acid sequences of different EV-G genotypes were downloaded from GenBank. The sequences were compared using MAFT Multiple Sequence Comparison software, and the similarity levels of nucleotide and amino acid sequences were calculated using the BioEdit software. Phylogenetic analysis was performed using the neighbor-joining method (Maximum Likelihood, NJ) using the MEGA software (Version 10.0.5) (15).

To analyze the recombination between the isolated strain and other known EV-G strains, their genome sequences were compared. Recombination analysis was performed using seven methods provided by the recombination analysis software “RDP-V4.1.3,” including RDP, GENECONV, BOOTSCAN, MaxChi, CHIMAERA, SISCAN, and 3Seq. When recombination signals were detected by three or more methods, the sequences were further analyzed using “Simplot-V3.5.1” (11).

### Pathogenicity of the isolated strain on 28-day-old piglets/experimental infection of piglets

Eight 28-day-old piglets that tested negative for EV-G, PEDV, TGEV, PRV-A, and PDCoV infections were purchased from a pig farm in Fuyuan County, Yunnan Province. The piglets were randomly assigned to two groups (groups A and B; four piglets per group) and housed in separate barns. After 3 days of adaptation, group A piglets were orally inoculated with 5 mL of the isolated strain (2 × 10^6^ PFU/mL), and group B piglets were inoculated with the same amount of BHK-21 cell culture supernatant to obtain the mock group (negative control). All eight piglets were fed three times a day and could drink water freely. The animals were clinically examined, and their body weights and rectal temperatures were monitored and recorded daily.

### Virus shedding and tissue virus load detection

An EV-G SYBR-Green RT-quantitative PCR (RT-qPCR) was performed to detect the viral load by targeting the 5′-UTR of the genome of the isolated strain (Table 1). The amplification product was cloned into the pMD19 vector (Takara Bio) to serve as the standard template and was serially diluted 10-fold to generate a standard curve. The primers were evaluated to ensure no cross-reactions with closely related PEVs.

Fecal samples were collected from 0 to 21 days post-infection (dpi). All piglets were then euthanized at 21 dpi, and their heart, liver, spleen, lungs, kidneys, brain, small intestine, mesenteric lymph nodes, and stomach were collected to detect the viral loads. Firstly, 0.5 g of each tissue or feces sample was homogenized with stainless steel beads of 2 mm diameter (EASYBIO, Beijing, China) in 1 mL sterile PBS using a TissueLyser II homogenizer (Qiagen, Hilden, Germany). The homogenized sample was then centrifuged at 8,000× g for 20 min at 4°C.

Virus RNA was extracted using a MiniBEST Viral RNA/DNA Extraction Kit Ver. 5.0 (Takara Bio) from 200 μL of the supernatant according to the manufacturer’s instructions. Subsequently, 10 μL of extracted RNA was used to synthesize cDNA using the PrimeScript™ 1^st^ Strand cDNA Synthesis Kit (Takara Bio). The RNA samples were reverse transcribed by sequentially incubating the samples at 30°C for 10 min, 42°C for 60 min, and 95°C for 5 min. Then, the samples were subjected to qPCR using the TB Green® Fast qPCR Mix Kit (Takara Bio) and a 7,500 Fast Real-Time PCR System (Applied Biosystems™). Ten-fold serially diluted (10^7^ to 10^1^ copies/μL) recombinant EV-G/YN/23/2029 plasmids were used to generate the standard curves to calculate the number of viral RNA copies in the animal samples.

### Histopathological examination

The brain, small intestine, and mesenteric lymph nodes of the piglets were fixed in 4% neutral-buffered formaldehyde (Servicebio, Wuhan, China) for 48 h and then dehydrated. The dehydrated samples were cleaned using xylene (Servicebio), embedded in paraffin wax (Sakura, Japan), and sectioned (5-μm thick) using a microtome (Shanghai Lycra, Shanghai, China). The sections were then mounted on slides and stained with hematoxylin and eosin (H&E staining; Servicebio) for histopathological observations using standard light microscopy (Nikon, Tokyo, Japan).

### Ethics statement

The study protocol was reviewed and approved by the Scientific Ethics Committee of YNASVI01-2022006. The animal experiments were conducted in biosafety level 2+ facilities at the Yunnan Biological Pharmaceutical Factory (Kunming, China).

## Results

### Sample analysis

Among the 32 pigs, five, three, and one samples tested positive for EV-G, PEDV, and a mixed PEDV and EV-G infection. All the pigs tested negative for TGEV, PoRV, and PDCoV.

### Isolation and identification of the isolated strain

**Figure 1:**
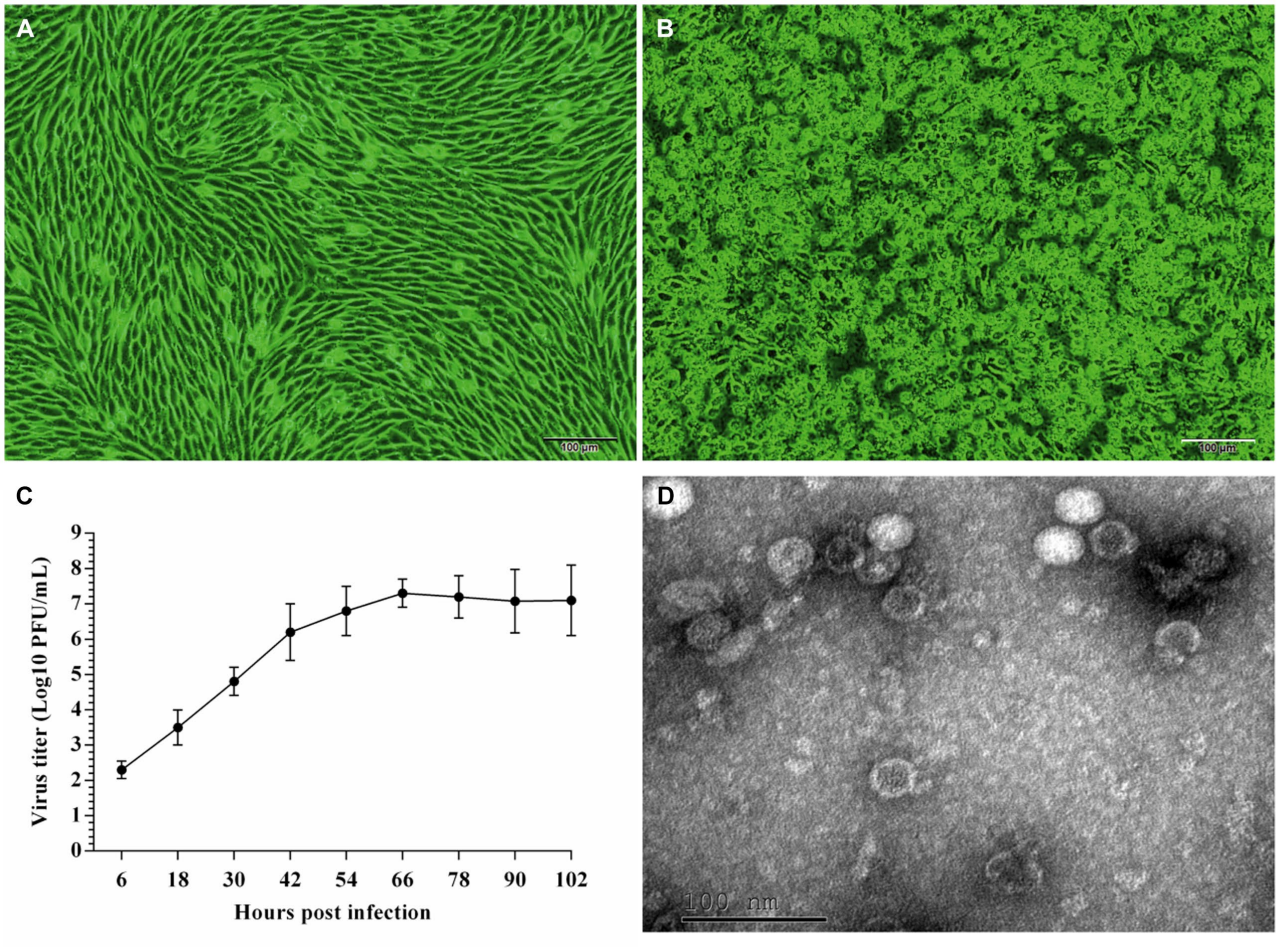
Cytopathic effects **(A,B)** and growth kinetics **(C)** of the EV-G/YN29/2022 strain in and the transmission electron microscopic image of purified EV-G particles **(D)**. **(A)** Uninfected BHK-21 cells; **(B)** BHK-21 cells 96 h post-infection; **(C)** Growth kinetics of EV-G/YN29/2022 in BHK-21 cells; **(D)** Transmission electron microscopic image of purified EV-G particles.

The five EV-G-positive samples were inoculated with BHK-21 cells. The supernatants containing the virus stocks were blindly passaged serially thrice and used to induce a severe CPE in BHK-21 cells, characterized by a distinct cell fusion, cell crumple, and gradual cell disintegration (Figures 1A,B). EV-G was detected from the culture supernatants using RT-PCR. Briefly, a specific ~500-bp long band was amplified, matching the expected size (492 bp). The sequencing of PCR products and online BLAST revealed that the nucleotide sequence of the isolated strain was 98% identical to that of the published EV-G sequence (PEV-B-KOR), indicating that the isolated virus was EV-G. This isolated strain was named “EV-G/YN29/2022.”

### Biological characteristics of the EV-G/YN29/2022 strain

The single plaque purification method was used to obtain purified virus samples. Virus plaques were morphologically homogeneous with clear contours. The titer of the purified viruses was 6.5 × 10^7^ PFU/mL. The proliferation curve indicated an initial rapid proliferation within 6 hpi, and peaked at 66 hpi (7.3 × 10^7^ PFU/mL). The virus stopped proliferating integration at 66 hpi, reaching a proliferative plateau by 102 hpi (Figure 1C). The virus titer at this stage was 7.1 × 10^7^ PFU/mL.

EV-G/YN29/2022 was purified, negatively stained, and observed through TEM to determine its morphological characteristics. We observed that the virus particles were nonenveloped and spherical, with an average diameter of 25–30 nm (Figure 1D).

### Sequence characterization of the EV-G/YN29/2022 strain

**Figure 2:**
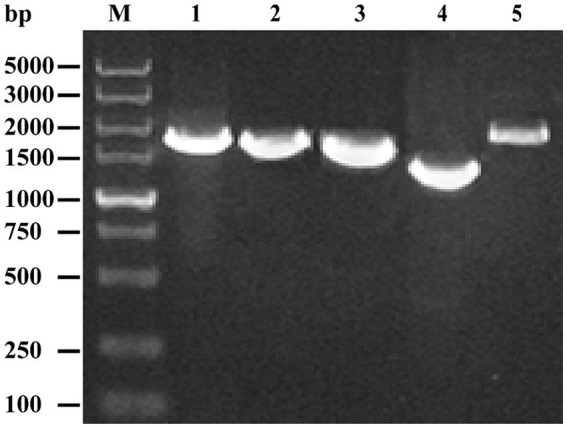
Amplification of the complete genome of the EV-G CH/17GXQZ/2017 strain using RT-PCR. Lane M: DNA marker DL 5000; Lanes 1~5: Five overlapping PCR products of the viral genome.

Five specific primer pairs were used to obtain the full genome sequence of the isolated EV-G strain. Our PCR-amplified product matched the expected size (Figure 2). The gene fragments were sequenced in the forward and reverse directions using M13F/R primers. The full genome sequence of the isolated strain was successfully obtained by assembling the sequencing results for each fragment, with the full genome length of 7,391 bp, GC content of 45.69%, and the length of the 5′-UTR region of 808 bp. The 3′ end, without the Poly A tail, was 68 bp long. The ORF region was 6,507 bp long, encoding a 2,169 amino acids long polyprotein precursor. Nucleotide sequence editing and analysis and amino acid sequence identification were performed using the EditSeq program in the DNASTAR package (DNASTAR Inc., United States). Sequences of all segments were subsequently submitted to the NCBI database with accession numbers PP681284.

### Phylogenetic analysis of the EV-G/YN29/2022 strain

**Figure 3:**
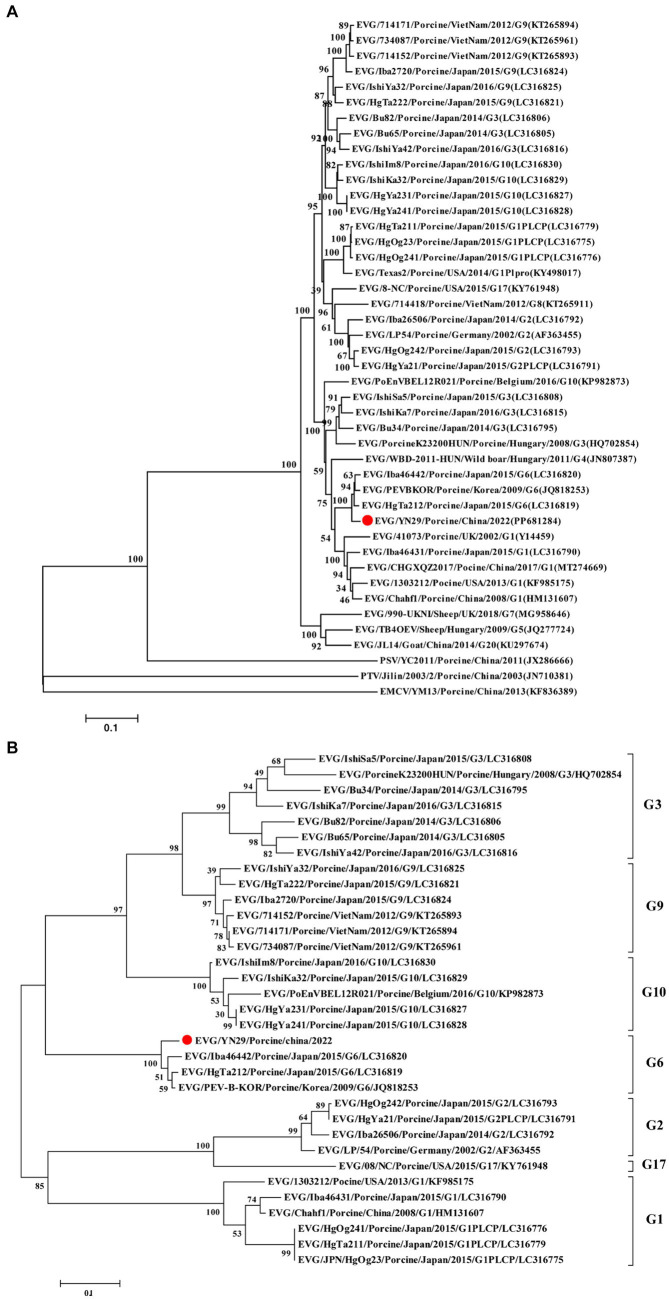
Phylogenetic tree of the complete genome sequences **(A)** and amino acid sequences of VP1 **(B)** of different EV-G strains using the maximum likelihood (ML) method. The EV-G/YN29/2022 strain is represented by a red dot, and other EV-G strains are indicated as “EV-G/Strain number/Host/Country/Year/Genotype/GenBank accession number”.

Comparison of the whole genome nucleotide sequences and their corresponding amino acid sequences showed that EV-G/YN29/2022 was most closely related to the EV-G6 genotypic strain, with nucleotide similarity and amino acid homology ranging from 83.7% and 96% (Iba46442) to 84.4% and 96.8% (PEV-B-KOR), respectively (Table 2). Phylogenetic analysis revealed that EV-G/YN29/2022 and EV-G6 clustered in one lineage (Figure 3A). Amino acid analysis revealed that both strains exhibited a high homology for the amino acid sequences of VP4, VP2, VP1, Pro, 2B, ATPase, and 3C proteins (Table 2). Of these, the amino acid sequences of the VPg protein were 100% similar between both strains, and the sequences of the VP3 and 3A proteins were highly similar (Table 2).

**Table 2:** Nucleotide and amino acid sequence identity (%) between EV-G/YN29/2022 and other EV-G-6 isolates listed in GenBank.

Gene	EV-G/YN29/2022		EV-G/Iba46442/Japan/2015 (LC316820)				EV-G/HgTa212/Japan/2015 (LC316819)				EV-G/PEV-B-KOR/Korea/2009 (JQ818253)			
	Size (nt)	Size (aa)	Size (nt)	Identity (%)	Size (aa)	Identity (%)	Size (nt)	Identity (%)	Size (aa)	Identity (%)	Size (nt)	Identity (%)	Size (aa)	Identity (%)
5′UTR	808	/	797	89.1	/	/	787	**92.2**	/	/	811	89.5	/	/
1A/VP4	207	69	207	81.7	69	89.8	207	**88.9**	69	95.6	207	85.0	69	**98.5**
2B/VP2	738	246	738	82.4	246	94.8	738	82.7	246	95.2	738	**83.1**	246	**95.6**
1C/VP3	831	277	831	**85.6**	277	**98.5**	831	83.4	277	97.8	831	83.5	277	97.5
1D/VP1	846	282	846	82.9	282	94.3	846	83.2	282	94.7	846	**84.2**	282	**95.4**
2A/Pro	450	150	450	82.6	150	94.2	450	**82.8**	150	94.9	450	81.8	150	**95.5**
2B	297	99	297	83.2	99	99.0	297	82.3	99	98.0	297	**85.8**	99	**100.0**
2C/ATPase	987	329	987	82.9	329	97.0	987	83.0	329	97.0	987	**85.6**	329	**97.6**
3A	267	89	267	**85.1**	89	**98.9**	267	79.3	89	97.8	267	82.6	89	97.8
3B/VPg	66	22	66	78.7	22	**100.0**	66	75.7	22	**100.0**	66	**83.3**	22	**100.0**
3C/Pro	549	183	549	**84.0**	183	**96.2**	549	81.7	183	95.7	549	82.7	183	**96.2**
3D/Pol	1,383	461	1,383	84.5	461	95.5	1,383	86.0	461	96.1	1,383	**86.8**	461	**96.5**
3′UTR	68	/	38	/	/	/	47	/	/	/	72	/	/	/
Total	7,391	2,169	7,341	83.7	2,169	96.0	7,344	84.0	96.3	2,169	7,393	**84.4**	2,169	**96.8**

nt, nucleotides; aa, amino acids; UTR, Untranslated region. Bold text shows the genes with the highest similarity with the EV-G/YN29/2022.

Further analyses showed that the VP1 gene sequence of EV-G/YN29/2022 and the corresponding amino acid sequence were highly similar to the corresponding sequences of EV-G6 (82.9% and 94.3%, respectively) but relatively less similar to the corresponding sequences of other EV-G genotypes, peaking at only 73.0% and 80.6%, respectively (Table 2). Moreover, phylogenetic tree analysis based on the amino acid sequence of the VP1-encoded protein showed that EV-G/YN29/2022 clustered with EV-G6 strains (Figure 3B), demonstrating that the isolated strain belonged to the EV-G6 genotype and was first isolated in China.

### Recombinant analysis of the EV-G/YN29/2022 genome

Recombinant analysis of the EV-G/YN29/2022 genome showed that the same recombination signal was detected when using MaxChi, CHIMAERA, and 3Seq (*p*-values: 1.948 × 10^−5^, 1.870 × 10^−2^, and 2.418 × 10^−4^, respectively). Furthermore, the fracture point commenced at 3551 and ended at 398, suggesting that EV-G/YN29/2022 might be a genetically recombinant strain, with PEV-B-KOR and 714,418/CaoLanh as the primary and secondary parental strains, respectively.

**Figure 4:**
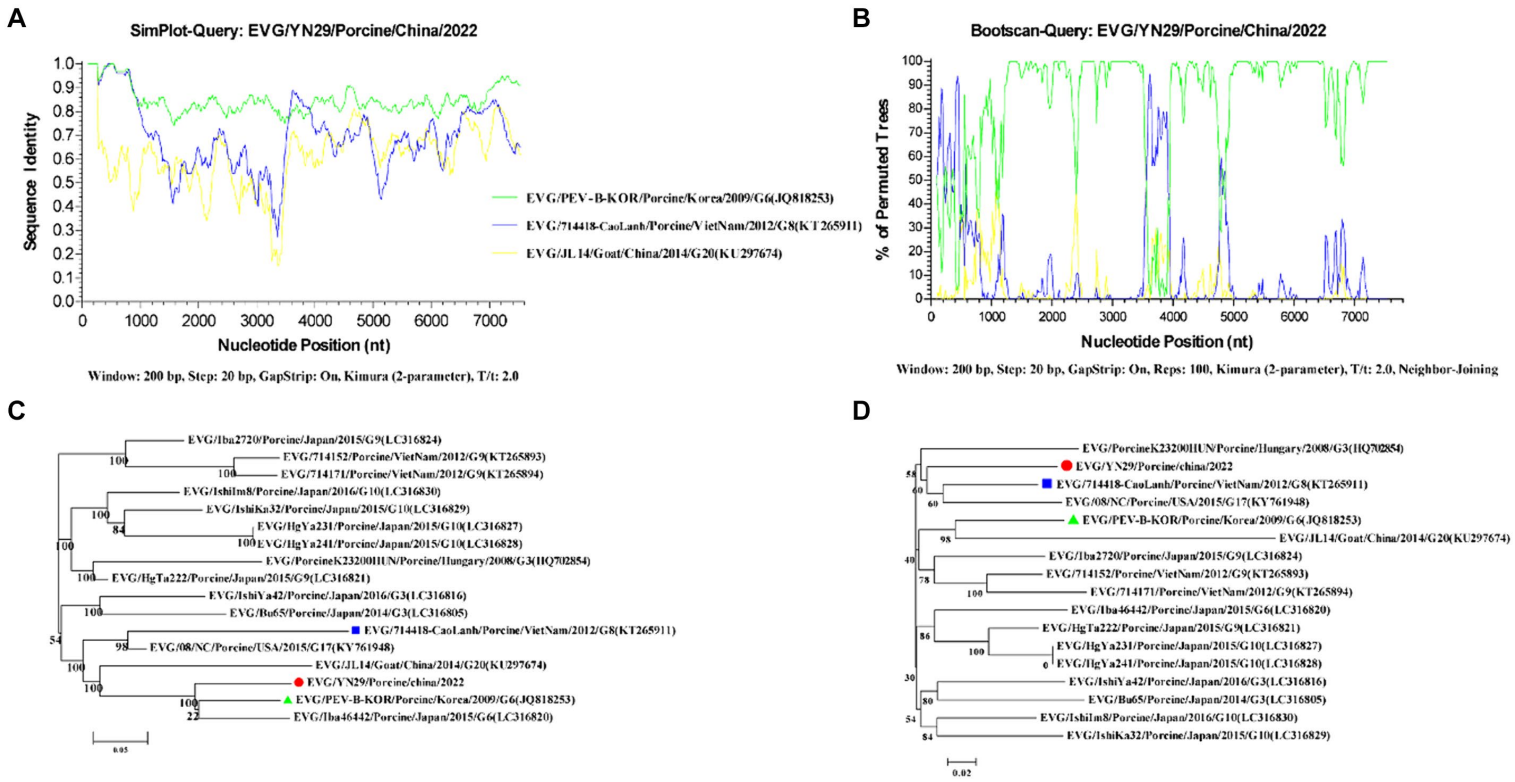
Recombination analyses of the genomes of EV-G/YN29/2022 and other EV-G strains. **(A)** Similarity analysis of the genome sequences of EV-G/YN29/2022 and its putative parents (EV-G/PEVBKOR/Porcine/Korea/2009/G6/JQ818253 and EV-G/714418/Porcine/VietNam/2012/G8/KT265911); **(B)** Boot scan analysis of the genome sequences of EV-G/YN29/2022 and its parents; **(C,D)**. Phylogenetic tree of different segments of the EV-G genome from positions 1–3,550 + 3,986–7,391 and positions 3,551–3,985 bp. The EV-G/YN29/2022 isolate is indicated by “●.” The major and minor putative parents are indicated by “▲” and “■,” respectively.

To verify the accuracy of the “recombination events,” similarities among the recombination sequences were analyzed using the “Simplot” software. Our results showed that positions 1 to 3,561 and 3,941 to 7,391 of the EV-G/YN29/2022 genomic sequence exhibited a high similarity (80%–100%) to the major parental strain (PEV-B-KOR) and a low similarity (26.9%–80%) to the minor parental strain (714,418/CaoLanh; Figure 4A). In contrast, positions 3,561 to 3,941 of EV-G/YN29/2022 were more similar to 714,418/CaoLanh (80–89%) and less similar to PEV-B-KOR (77%–83%; Figure 4A). Boot scan analysis of the sequences of the EV-G/YN29/2022 strain and its parental strains revealed recombination signals between positions 3,561 and 3,941 of the EV-G/YN29/2022 genome, with permuted trees values ranging from 55.5% to 94.6% (Figure 4B).

Furthermore, phylogenetic trees were constructed based on the sequences in tandem in the regions before and after the reorganization breakpoint (bits 1 to 3,550 and 3,986 to 7,391) and the sequences in the region within the breakpoint (bits 3,551 to 3,985). Our results showed that in the tandem region, EV-G/YN29/2022 exhibited the closest affinity to PEV-B-KOR and was more distant from 714,418/CaoLanh (Figure 4C). In contrast, in the middle region of the breakpoint (positions 3,551 to 3,985), EV-G/YN29/2022 exhibited the closest affinity to 714,418/CaoLanh and was farther from PEV-B-KOR (Figure 4D). These findings suggested that EV-G/YN29/2022 was a recombinant strain, with the major and minor parental strains being PEV-B-KOR and “714,418/CaoLanh,” respectively.

### Clinical examination

To assess the pathogenicity of the isolated strain, 28-day-old piglets were orally inoculated with 5 mL of either the EV-G/YN29/2022 solution (group A) or BHK-21 cell culture supernatant (group B). All piglets exhibited normal body temperatures at 0–21 dpi. Group A piglets exhibited anorexia at 5–8 dpi, mild diarrhea at 6–9 dpi, and yellow pasty feces. However, the diarrhea symptoms gradually alleviated after 12 dpi. Moreover, the infected piglets exhibited skin rashes at 7–12 dpi, which gradually disappeared at 13–15 dpi. None of these clinical symptoms were observed in group B piglets. None of the infected piglets died, indicating that EV-G/YN29/2022 is mildly pathogenic.

### Necropsy and histopathological analysis

**Figure 5:**
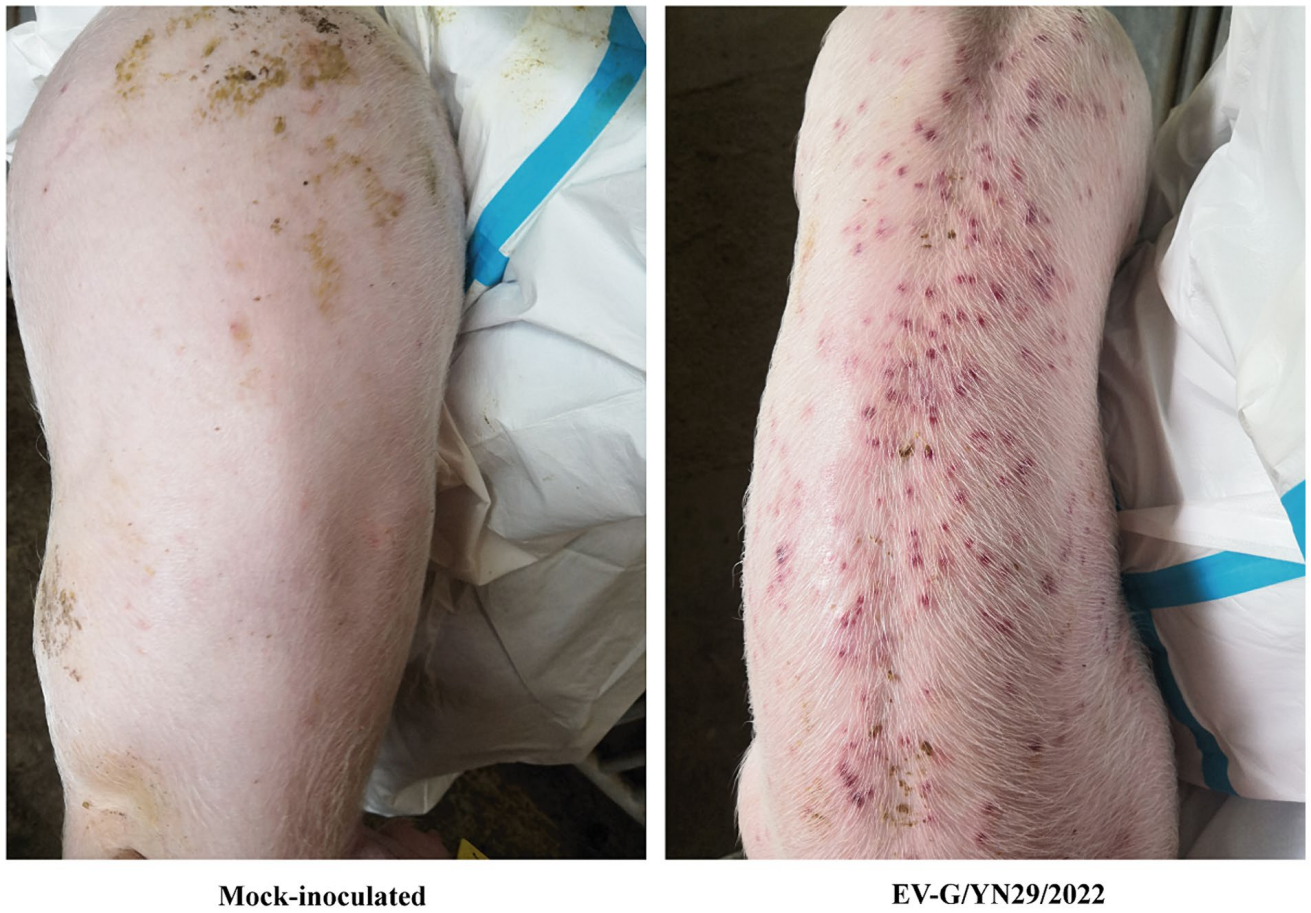
The skin rashes of the EV-G/YN29/2022 challenged piglets.

**Figure 6:**
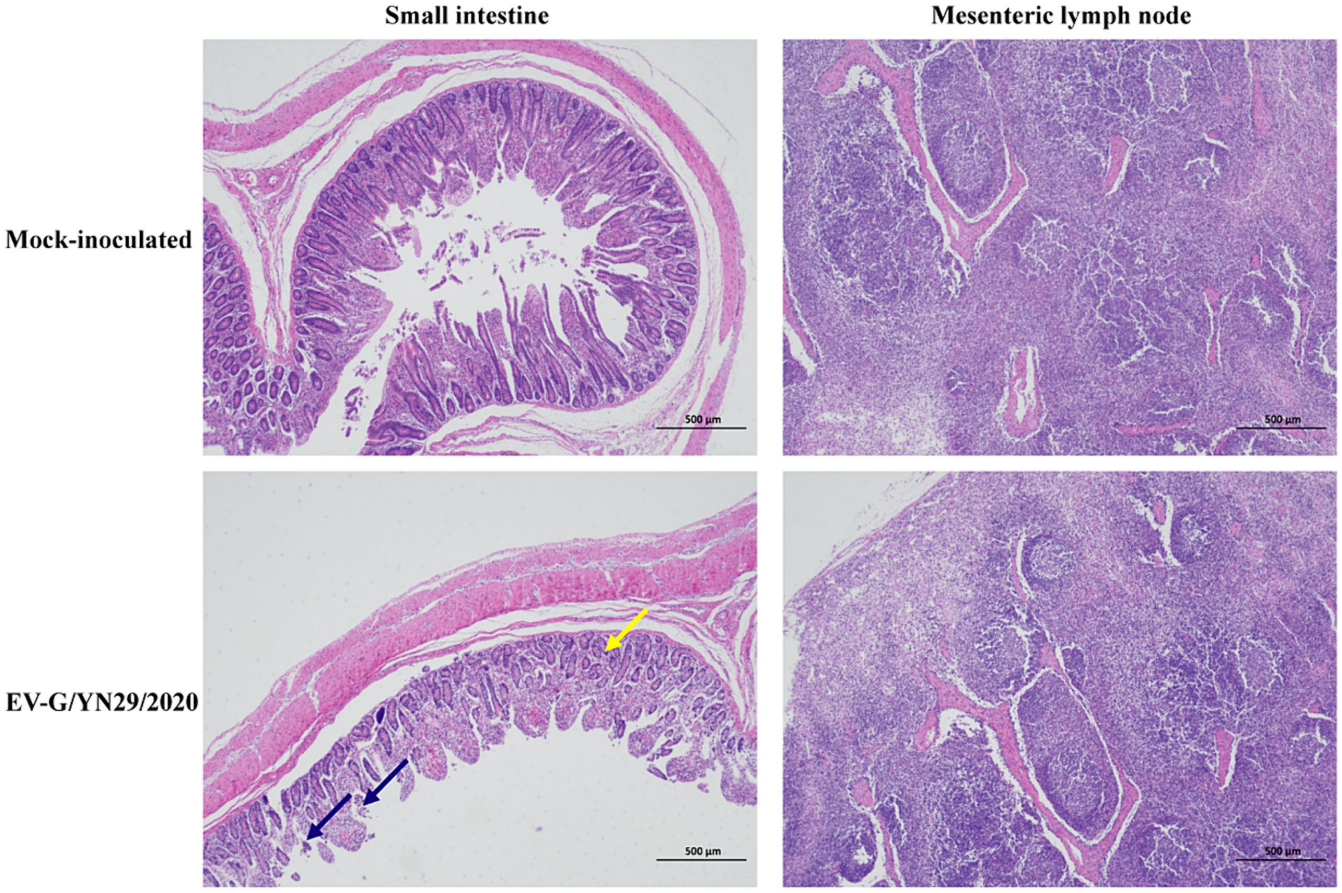
Histopathological analysis of the piglets challenged with EV-G/YN29/2022 and control piglets. Hematoxylin and eosin (H&E) staining of the small intestine tissues showed local exfoliation and defects in the epithelial mucosae (blue arrow) and the irregularly arranged and enlarged space of the lamina propria (yellow arrow) in the infected pigs (Scale bar = 500 μm).

All pigs were euthanized and necropsied at 21 dpi. We observed mild hemorrhaging in the small intestine and mesenteric lymph nodes of the infected piglets. Histopathological analysis of the small intestine showed that some intestinal villi were necrotic, and the epithelial mucosae exhibited local exfoliation and defects (Figure 5). Furthermore, the lamina propria was arranged less regularly in the infected group compared to the mock group (Figure 6). In contrast, no pathological lesions were observed in the mesenteric lymph nodes of the infected piglets.

### Viral shedding and distribution

**Figure 7:**
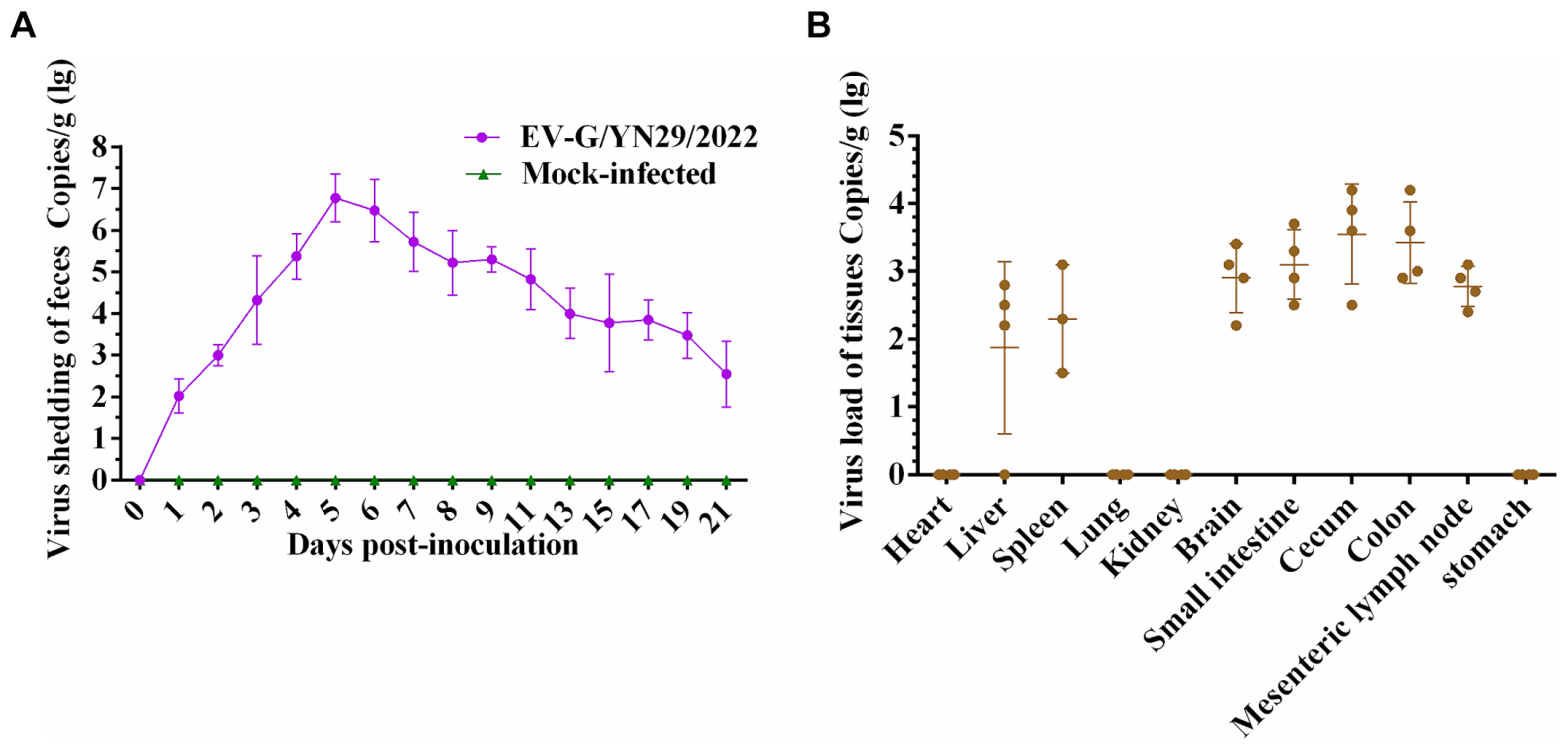
Viral RNA copies in fecal swab, heart, liver, spleen, lungs, kidneys, brain, small intestine, cecum, colon, mesenteric lymph nodes, and stomach of the piglets challenged with EV-G/YN29/2022. **(A)** Fecal virus shedding in the infected piglets from 0 to 21 days post-infection. **(B)** Virus loads in different organs of the infected piglets at 21 days post-infection.

Fecal swab samples of all piglets were collected from 0 to 21 dpi, and viral copies were detected using RT-qPCR. Our results showed that viral copies of EV-G increased rapidly between 12 and 48 hpi, peaking at 5 dpi with a titer of 10^6.77^ copies/g. Then, virus shedding gradually decreased but still remained detectable at the end of the experiment (Figure 7A). In the infected group, EV-G viral copies were detected in the brain, small intestine, mesenteric lymph nodes, spleen, and liver, but no viral RNA was detected in the heart, lungs, stomach, and kidneys. These findings indicated that the cecum and colon of the infected piglets harbored more EV-G copies than their small intestine (Figure 7B).

## Discussion

Currently, EV-Gs have been reported in the United States, Europe, and Asia (16). In China, EV-G was first isolated in Shanghai in 2012, followed by successive isolations from pigs in Anhui, Jiangsu, and Guangxi provinces. Most of the isolated EV-G strains were detected from the feces of swine, indicating that EV-G was the potential causal agent for porcine diarrhea. Our retrospective investigation indicated a high prevalence of natural EV-G infections in pig herds.

Previously, Taveesak Janetanakit et al. (1) conducted a cross-sectional survey of EV-G infection in pigs from 73 pig farms in 20 provinces of Thailand. Their results showed a high EV-G prevalence, with 71.6% of fecal and intestinal samples (556/777) and 71.2% of pig farms (52/73) positive for EV-G infection. Similarly, Vilar et al. (17) detected a high EV-G prevalence in the fecal samples of clinically healthy pigs in Catalonia, Spain. In another study, Yang et al. (18) investigated the prevalence of PEV-9 in pigs in the middle and eastern regions of China, reporting an overall infection prevalence of 8.3% (37/447) in the studied pigs. These results showed no significant differences between the EV-G prevalences in the fecal samples of diarrheic and healthy pigs, indicating no direct correlation between EV-G prevalence and porcine diarrhea.

In the current study, we detected PEDV infection in samples harboring EV-G/YN29/2022. EV-G can be propagated in BHK-21, Vero, ST, and Marc-145 cells (19). The first EV-G strain which was isolated in the United States used Marc-145 cells until 2013 (12). EV-G-PLCP recombinant viruses were originally obtained using ST cells in 2015 (20). Cytopathic effects and growth kinetics on BHK-21 cells trend to be similar to that described previously, and the virus titer reaching its peak at 66 hpi. PEDV is the most dominant virus found in diarrheic animals. Corroborating our results, some previous studies have also detected multiple RNA viruses in the fecal samples from PEDV-infected pigs (21), suggesting that PEDV might synergistically infect the animals harboring multiple RNA viruses. This finding warrants an in-depth study of the relationship between EV-G and other intestinal pathogens.

Previous studies have reported varying EV-G prevalence with piglet age, with the incidence being significantly higher in younger pigs (nursery and fattening piglets) than in adult pigs. In the current study, we detected EV-G-positive samples in five out of the 32 piglets, including one weaned pig, two nursery pigs, one fattening pig, and one reserve pig. This finding was consistent with the results of the previous studies. Furthermore, the strain isolated in the current study exhibited the EV-G6 genotype. Notably, this is the first report of the isolation of a G6 genotype strain in China.

The VP1 protein of EV-G is located in the surface layer of the viral capsid and is the main component determining the antigenicity of the virus (22). Compared to the other viral proteins, the nucleotide and corresponding amino acid sequences of the VP1 gene are more diverse across different EV-G strains (5). Currently, EV-G strains are categorized into distinct genotypes (EV-G1 to EV-G20) based on the differences in the VP1 nucleotide sequence and corresponding amino acid sequence (5, 6). Strains with less than 75 and 82% similarities in nucleotide and amino acid sequences were defined as different genotypes. In the current study, the VP1 gene sequence (82.9%), full genome sequence (83.7%), and the encoded amino acid sequences (94.3% and 96%, respectively) of the EV-G/YN29/2022 strain were more similar to the corresponding sequences of the EV-G6 genotype than other genotypes. These results indicated the EV-G/YN29/2022 strain belonged to the EV-G6 genotype.

Genetic recombination between different EV strains is common and plays an important role in the evolution of EVs (23). This recombination might be associated with cross-species transmission of EVs, the capability of the virus to evade the host immune system, and changes in the virulence and pathogenicity of the virus (9, 24). Genetic recombination is also common between different EV-G strains. One type of recombination occurs in the 5′-UTR region of the EV-G genome and is termed as “recombination type 1” (4). Another type of recombination entails the insertion of the PLCP gene of ToV between the “2C” and “3A” regions of the EV-G genome (25). A previous study showed that the ToV-PLCP gene could potentially suppress the host cellular innate immune response and act as an “innate immune antagonist,” thus altering the pathogenicity of EV-G when inserted into its genome (26). We detected recombination signals between genomic positions 3,561 and 3,941 (partial sequences of genes 2A and 2B); however, it is unclear whether this recombination event represents a novel recombination type in the genome of the EV-G strains. Further studies are needed to identify the factors that impact the pathogenicity of an EV-G strain post-recombination.

Our previous study showed that the pathogenicity of EV-G was comparable to that of EV-G-PLCP (27). Recent research found EV-G may not been widely associated with diarrhea in swine, except for single reports of skin lesions, flaccid paralysis (18), the high prevalence and large number of current genotypes may suggest that EV-G-mediated pathogenesis requires confounding factors (e.g., co-infections), or that only certain genotypes trigger clinical manifestation (8). Some enteroviruses have been shown to have the ability to infect the central nervous system and cause various effects such as paralysis and ataxia (28). In our study, piglets infected with the EV-G/YN29/2022 strain showed no clinical neurological symptoms, but found virus loads in brain of challenged piglets at 21 dpi, which indicated EVs do not always produce neurological symptoms when they enter the brain.

## Conclusion

In this study, an EV-G6 strain was isolated and purified for the first time from pigs in the Yunnan province. The whole genome of the isolated virus was sequenced. The pathogenicity of the EV-G/YN29/2022 strain was determined in 31-day-old piglets. Our results could be used as a reference for further elucidating the biological characteristics, evolution, and pathogenicity of EV-G.

## Data availability statement

The data presented in the study are deposited in the NCBI repository, accession number PP681284.

## Ethics statement

The animal study protocol was approved by the Yunnan Animal Science and Veterinary Institute with the approval number YNASVI01-2022006. The study was conducted in accordance with the local legislation and institutional requirements.

## Author contributions

PZ: Conceptualization, Data curation, Formal analysis, Writing – original draft, Writing – review & editing. Z-HL: Conceptualization, Data curation, Writing – review & editing. Z-RL: Investigation, Software, Writing – review & editing. Z-XZ: Resources, Supervision, Writing – review & editing. J-LS: Funding acquisition, Project administration, Resources, Writing – review & editing.
