# Supplementary material for: First isolation, identification, and pathogenicity evaluation of an EV-G6 strain in China
Source: Front Vet Sci. 2024 Jul 24;11:1431180. doi: 10.3389/fvets.2024.1431180 (PMC11304196; doi:10.3389/fvets.2024.1431180)
Supplement: Supplementary file 1 [file Table_1.DOCX]

Table1 Fecal virus shedding in EV-G/YN29/2029-challenged piglets from 0 to 21 dpi（Copies/g (lg).

| Day | Chanllenged | | | |  | Mock inoculated | | |
| --- | --- | --- | --- | --- | --- | --- | --- | --- |
|  | pig1 | pig2 | pig3 | pig4 |  | pig1 | pig2 | pig3 |
| 0 | 0.0 | 0.0 | 0.0 | 0.0 |  | 0.0 | 0.0 | 0.0 |
| 1 | 2.5 | 2.0 | 1.5 | 2.1 |  | 0.0 | 0.0 | 0.0 |
| 2 | 2.9 | 3.3 | 2.7 | 3.1 |  | 0.0 | 0.0 | 0.0 |
| 3 | 3.8 | 5.4 | 3.1 | 5.0 |  | 0.0 | 0.0 | 0.0 |
| 4 | 5.5 | 6.1 | 4.9 | 5.0 |  | 0.0 | 0.0 | 0.0 |
| 5 | 6.1 | 7.5 | 6.8 | 6.7 |  | 0.0 | 0.0 | 0.0 |
| 6 | 5.5 | 6.9 | 7.2 | 6.3 |  | 0.0 | 0.0 | 0.0 |
| 7 | 4.9 | 6.6 | 5.9 | 5.5 |  | 0.0 | 0.0 | 0.0 |
| 8 | 4.5 | 6.3 | 5.2 | 4.9 |  | 0.0 | 0.0 | 0.0 |
| 9 | 5.0 | 5.7 | 5.2 | 5.3 |  | 0.0 | 0.0 | 0.0 |
| 11 | 4.9 | 4.5 | 5.8 | 4.1 |  | 0.0 | 0.0 | 0.0 |
| 13 | 3.6 | 3.8 | 4.9 | 3.7 |  | 0.0 | 0.0 | 0.0 |
| 15 | 3.9 | 3.4 | 5.3 | 2.5 |  | 0.0 | 0.0 | 0.0 |
| 17 | 3.8 | 4.1 | 4.3 | 3.2 |  | 0.0 | 0.0 | 0.0 |
| 19 | 3.5 | 3.8 | 3.9 | 2.7 |  | 0.0 | 0.0 | 0.0 |
| 21 | 3.0 | 2.4 | 1.5 | 3.3 |  | 0.0 | 0.0 | 0.0 |

Table2 Virus loads in different organs of EV-G/YN29/2020-challenged piglets at 21 dpi（Copies/g (lg).

| organs | Chanllenged | | | |  | Mock inoculated | | |
| --- | --- | --- | --- | --- | --- | --- | --- | --- |
|  | pig1 | pig2 | pig3 | pig4 |  | pig1 | pig2 | pig3 |
| Heart | 0 | 0 | 0 | 0 |  | 0.0 | 0.0 | 0.0 |
| Liver | 2.5 | 2.2 | 0 | 2.8 |  | 0.0 | 0.0 | 0.0 |
| Spleen | 1.5 | 2.3 |  | 3.1 |  | 0.0 | 0.0 | 0.0 |
| Lung | 0 | 0 | 0 | 0 |  | 0.0 | 0.0 | 0.0 |
| Kidney | 0 | 0 | 0 | 0 |  | 0.0 | 0.0 | 0.0 |
| Brain | 3.4 | 3.1 | 2.2 | 2.9 |  | 0.0 | 0.0 | 0.0 |
| Small intestine | 2.9 | 3.3 | 2.5 | 3.7 |  | 0.0 | 0.0 | 0.0 |
| Cecum | 3.9 | 3.6 | 2.5 | 4.2 |  | 0.0 | 0.0 | 0.0 |
| Colon | 4.2 | 2.9 | 3 | 3.6 |  | 0.0 | 0.0 | 0.0 |
| Mesenteric lymph node | 2.7 | 2.4 | 3.1 | 2.9 |  | 0.0 | 0.0 | 0.0 |
| stomach | 0 | 0 | 0 | 0 |  | 0.0 | 0.0 | 0.0 |

Table3 daily weight gain（kg/day）

| Infection time/day | EVG**-29 (n=4)** | | | |  | Con**trol (n=3)** | | |
| --- | --- | --- | --- | --- | --- | --- | --- | --- |
| 1 | 0.6 | 0.5 | 0.3 | 0.4 | 0.8 | 0.7 | 0.5 | 0.4 |
| 2 | 0.4 | 0.7 | 0.5 | 0.7 | 0.4 | 0.7 | 0.8 | 0.4 |
| 3 | 0.6 | 0.4 | 0.7 | 0.7 | 0.6 | 0.6 | 0.8 | 0.4 |
| 4 | 0.5 | 0.7 | 0.8 | 0.3 | 0.4 | 0.8 | 0.4 | 0.5 |
| 5 | 0.3 | 0.5 | 0.9 | 0.5 | 0.3 | 0.7 | 0.5 | 0.6 |
| 6 | 0.5 | 0.3 | 0.8 | 0.6 | 0.3 | 0.7 | 0.4 | 0.4 |
| 7 | 0.6 | 0.4 | 1.2 | 0.3 | 0.4 | 0.5 | 0.7 | 0.6 |
| 8 | 0.9 | 0.5 | 0.8 | 0.7 | 0.2 | 0.7 | 0.6 | 0.6 |
| 9 | 0.6 | 0.6 | 0.5 | 0.4 | 0.6 | 0.4 | 0.7 | 0.5 |
| 10 | 0.7 | 0.9 | 0.4 | 0.6 | 0.7 | 0.4 | 0.5 | 0.6 |
| 11 | 0.8 | 0.5 | 0.9 | 0.6 | 0.4 | 0.5 | 0.8 | 0.8 |
| 12 | 0.5 | 0.9 | 0.4 | 0.5 | 0.4 | 0.5 | 0.7 | 0.6 |
| 13 | 0.5 | 0.7 | 0.4 | 0.2 | 0.5 | 0.8 | 0.5 | 0.4 |
| 14 | 0.4 | 0.8 | 0.5 | 0.5 | 0.4 | 0.5 | 0.9 | 0.3 |
| 15 | 0.7 | 0.8 | 0.6 | 0.6 | 0.5 | 0.6 | 0.2 | 0.5 |
| 16 | 0.6 | 0.5 | 0.3 | 0.8 | 0.6 | 0.4 | 0.8 | 0.7 |
| 17 | 0.5 | 0.7 | 0.4 | 0.5 | 0.4 | 0.5 | 0.5 | 0.8 |
| 18 | 0.3 | 0.9 | 0.7 | 0.6 | 0.7 | 0.6 | 0.8 | 0.5 |
| 19 | 0.7 | 0.8 | 0.4 | 1 | 0.7 | 0.6 | 0.8 | 0.7 |
| 20 | 0.8 | 0.6 | 1.1 | 0.5 | 0.3 | 0.9 | 0.6 | 0.5 |
| 21 | 1.0 | 0.8 | 0.6 | 0.7 | 0.3 | 0.6 | 0.5 | 0.5 |
